# Drug allergy

**DOI:** 10.1186/s13223-024-00936-1

**Published:** 2025-01-22

**Authors:** Samira Jeimy, Tiffany Wong, Moshe Ben-Shoshan, Ana Maria Copaescu, Ghislaine A. C. Isabwe, Anne K. Ellis

**Affiliations:** 1 Division of Clinical Immunology and Allergy, Department of Medicine, Western University, London, ON Canada; 2 Division of Allergy, Department of Pediatrics, The University of British Columbia, Vancouver, BC Canada; 3 Division of Pediatric Allergy Clinical Immunology and Dermatology, Department of Pediatrics, McGill University Health Center, Montreal, QC Canada; 4 Division of Allergy and Clinical Immunology, Department of Medicine, McGill University Health Centre (MUHC), McGill University, Montreal, QC Canada; 5 The Research Institute of the McGill University Health Centre, , McGill University, McGill University Health Centre (MUHC), Montreal, QC Canada; 6 Department of Infectious Diseases, Centre for Antibiotic Allergy and Research, Austin Health, Heidelberg, VIC Australia; 7 Division of Allergy & Immunology, Department of Medicine, Queen’s University, Kingston, ON Canada

## Abstract

Drug allergy encompasses a spectrum of immunologically-mediated hypersensitivity reactions (HSRs) with varying mechanisms and clinical presentations. This type of adverse drug reaction (ADR) not only affects patient quality of life, but may also lead to delayed treatment, unnecessary investigations, and increased morbidity and mortality. Given the spectrum of symptoms associated with the condition, diagnosis can be challenging. Therefore, referral to an allergist experienced in the diagnosis and management of drug allergy is recommended if a drug-induced allergic reaction is suspected. Diagnosis relies on a careful history and physical examination and, in some instances, skin testing or in vitro testing and drug challenges. The most effective strategy for the management of allergist-confirmed drug allergy is avoidance or discontinuation of the offending drug. When available, alternative medications with unrelated chemical structures should be substituted. Cross-reactivity among drugs should also be taken into consideration when choosing alternative agents. Additional therapy for drug HSRs may include topical corticosteroids, oral antihistamines and, in severe cases, systemic corticosteroids and other immunomodulators. In the event of anaphylaxis, the treatment of choice is intramuscular epinephrine. If a patient with a history of anaphylaxis requires a specific drug and there is no acceptable alternative, desensitization to that drug may be considered. This article provides a background on drug allergy and strategies for the diagnosis and management of some of the most common drug-induced allergic reactions.

## Key take-home messages

- Drug allergy encompasses a spectrum of immunologically mediated hypersensitivity reactions (HSRs) with varying mechanisms and clinical presentations.
- Risk factors for drug allergy include age (more common in young/middle-aged adults), gender (more common in females), genetic polymorphisms, certain viral infections (HIV and herpes viruses) and drug-related factors (topical and IV/intramuscular routes of administration are more immunogenic than oral administration).
- The skin is the organ most frequently affected by drug-induced allergic reactions; however, many other organ systems may be involved, including multi-organ reactions such as anaphylaxis.
- Referral to an allergist is important for the appropriate diagnosis and treatment of drug allergy.
- Diagnosis requires a thorough drug history, including dates of administration, drug formulation, dosage and route of administration, as well as clinical symptoms and their timing and duration in relation to drug exposure; skin testing and graded challenges may also be required.
- The mainstay of treatment is avoidance of the offending drug; alternative medications with unrelated chemical structures should be substituted when possible.
- If a particular drug to which the patient is allergic is indicated, induction of drug tolerance procedures may be considered to induce temporary tolerance to the drug.

## Introduction

An adverse drug reaction (ADR) is defined as a harmful or unintended reaction to a drug that occurs at doses used for prevention, diagnosis, or treatment [1, 2]. ADRs are common in everyday clinical practice, affecting 15–25% of patients; serious reactions occur in 7–13% of patients [3, 4]. ADR can be classified into two broad categories, as described in Table 1 [1, 5, 6].

**Table 1:** Classification of ADRs [1, 5, 6]

ADR type	Characteristics	Examples
A	• Common • Predictable—may occur in anyone • Dose dependent • Related to known pharmacologic actions of drug	• Drug overdose • Secondary drug effects • Side effects • Drug interactions
B	• 20–25% of ADRs • Unpredictable • Not necessarily dose dependent • Unrelated to known pharmacologic actions of drug	• Drug allergy: immunologically mediated, 5–10% of ADRs • *Non-IgE-mediated reactions (previously called pseudoallergic or anaphylactoid)*: a reaction with the same clinical manifestations as an allergic reaction, but that lacks immunological specificity • *Drug intolerance*: an undesirable pharmacologic effect that occurs at low and sometimes sub-therapeutic doses of the drug that are not caused by underlying abnormalities of metabolism or drug excretion • *Drug idiosyncrasy*: an abnormal/unexpected effect, usually caused by underlying abnormalities of metabolism, excretion, or bioavailability

ADRs: adverse drug reactions; IgE: immunoglobulin E

ADRs can also be conceptualized as immediate or non-immediate/delayed, based on their latency from exposure to symptom onset. Immediate drug reactions are more likely to be “true allergies” (immunoglobulin E [IgE]-mediated), and typically occur within 1 h (up to 6 h) after drug administration [7–9]. Non-immediate or delayed reactions occur after 6 h of the initial drug administration.

A Drug allergy is an immunologically-mediated type B ADR (Table 1) that not only affects patient quality of life, but may also lead to delayed treatment, use of suboptimal alternate medications, unnecessary investigations, and increased morbidity and mortality. Furthermore, the identification of a drug allergy is challenging given the myriad of symptoms and clinical presentations associated with the condition. Therefore, if a drug allergy is suspected, consultation with an allergist experienced in the identification, diagnosis and management of drug allergy is recommended. This article will provide an overview of the mechanisms and risk factors for drug allergy, as well as strategies for the diagnosis and appropriate management of some of the most common drug-induced allergic disorders.

## Mechanisms

**Fig. 1:**
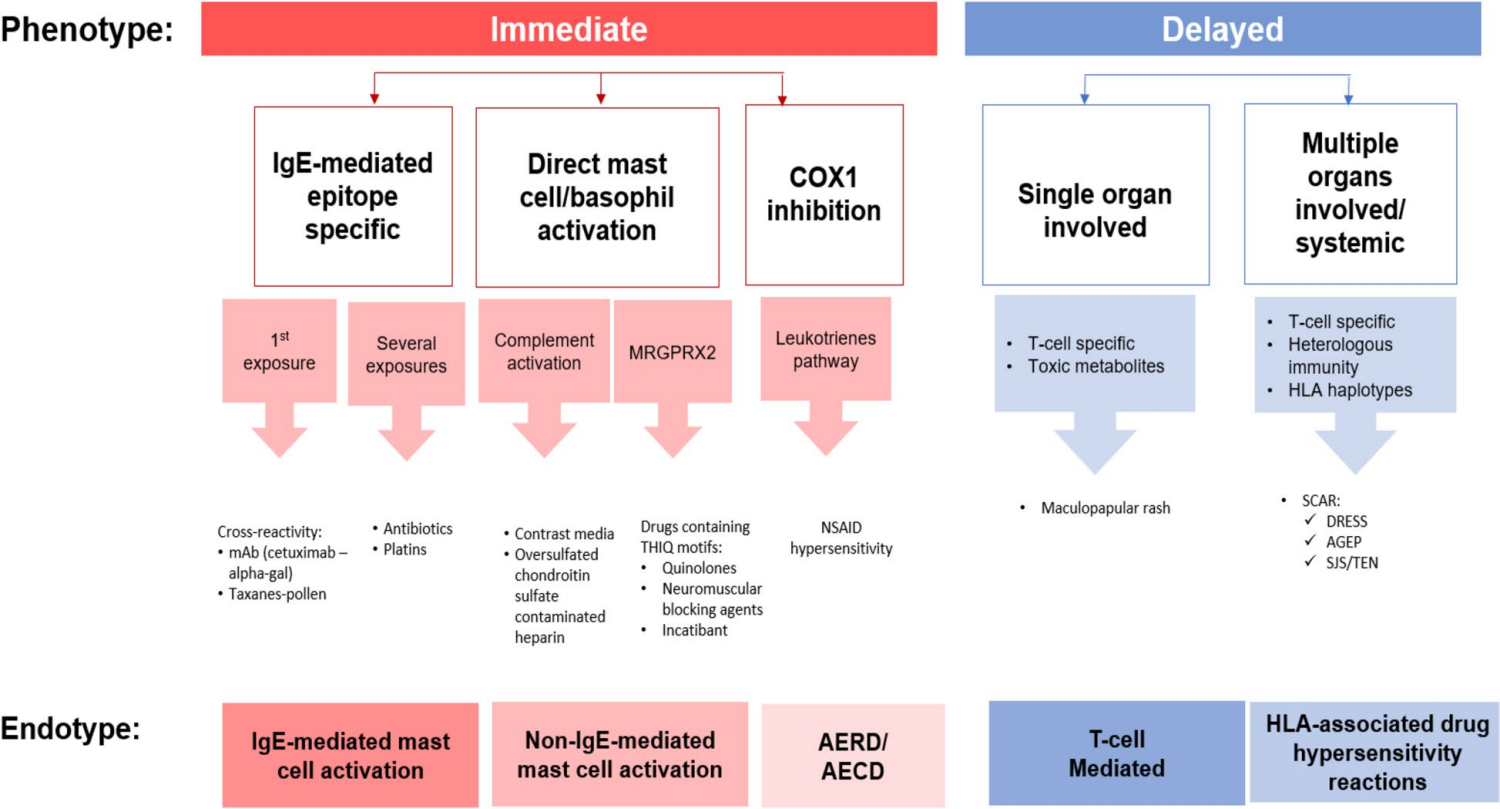
Drug allergy classification based on phenotypes and endotypes [9, 14]. AECD: aspirin-exacerbated cutaneous disease; AERD: aspirin-exacerbated respiratory disease; AGEP: acute generalized exanthematous pustulosis; alpha-gal: galactose-alpha-1,3-galactose; COX1: cyclooxygenase-1; DRESS: drug reaction with eosinophilia and systemic symptoms; HLA: human leukocyte antigen; IgE: immunoglobulin E; mAb: monoclonal antibody; MRGPRX2: Mas-related G protein-coupled receptor; NSAID, non-steroidal anti-inflammatory drug; SJS: Stevens-Johnson syndrome; TEN: toxic epidermal necrolysis. Figure adapted from: de Las Vecillas Sánchez et al. [14] and Muraro et al. [9]

Immune-mediated allergic reactions to drugs are divided according to the Gell and Coombs’ classification system, which describes the predominant immune mechanisms involved in these reactions. This classification system includes: immediate-type reactions mediated by IgE antibodies (type I), cytotoxic reactions mediated by immunoglobulin G (IgG) or M (IgM) antibodies (type II), immune-complex reactions (type III), and delayed-type SRs mediated by cellular immune mechanisms, such as the recruitment and activation of T cells (type IV) [10–13]. The mechanisms, clinical manifestations, and timing of these immune reactions are summarized in Table 2. A more recent drug allergy classification based on phenotypes and endotypes has been proposed by Muraro et al. (see Fig. 1) [9, 14].

**Table 2:** Classification of allergic drug reactions: mechanisms, clinical manifestations, and timing of reactions [10–13]. Adapted from Riedl et al. [10]

Immune reaction	Mechanism	Clinical manifestations	Timing of reaction
Type I (IgE-mediated)	Drug-IgE complex binding to mast cells with release of histamine, inflammatory mediators	Anaphylaxis^a^, urticaria^a^, angioedema^a^, bronchospasm^a^	Minutes to hours after drug exposure
Type II (cytotoxic)	Specific IgG or IgM antibodies directed at drug-hapten coated cells	Anemia, cytopenia, thrombocytopenia	Variable
Type III (immune complex)	Tissue deposition of drug-antibody complexes with complement activation and inflammation	Serum sickness, vasculitis, fever, rash, arthralgia	1 to 3 weeks after drug exposure
Type IV (delayed, cell mediated)^b^	MHC presentation of drug molecules to T cells with cytokine and inflammatory mediator release; may also be associated with activation and recruitment of eosinophils, monocytes, and neutrophils	Contact dermatitis, delayed morbilliform reactions, organ damage	2 to 7 days after drug exposure; can be up to 8 weeks

AGEP: acute generalized exanthematous pustulosis; DRESS: drug reaction with eosinophilia and systemic symptoms; IgE: immunoglobulin E; IgG: immunoglobulin G; IgM: immunoglobulin G; MHC: major histocompatibility complex

^a^These reactions may also be non-immunologically mediated

^b^Type IV reactions can be further classified into the following subtypes: type IVa which involve macrophages (e.g., contact dermatitis); type IVb which involve eosinophils (e.g., DRESS syndrome); type IVc which involve CD4 + or CD8 + T cells (e.g., maculopapular); and type IVd which involve neutrophils (e.g., AGEP)

There are several theories to explain how a low molecular weight compound such as a drug is able to stimulate an immune response: (1) the hapten hypothesis; (2) the pharmacological-interaction (p-i) hypothesis [15]; (3) the direct mast cell activation hypothesis [16] and (4) the altered peptide repertoire model [17]. In the hapten theory, the drug binds to a ubiquitous, larger molecular weight serum protein (e.g., serum albumin). This drug-self-protein combination is processed by antigen-presenting cells (APCs) of the immune system and presented to T cells that recognize the modified self-protein. The p-i hypothesis proposes that the drug binds to a cell-surface receptor, such as the major histocompatibility complex (MHC) or the T-cell receptor, and modifies its structure so that it is recognized by other cells of the adaptive immune system as foreign, thereby stimulating an immune response. The third hypothesis entails the direct activation of mast cells through a receptor called Mas-related G protein-coupled receptor X2 (MRGPRX2), essentially mimicking an allergic reaction without involving specific antibodies [16]. Finally, the altered peptide repertoire hypothesis suggests that a drug can interact with human leukocyte antigen (HLA) class I molecules in a specific and noncovalent manner, leading to the presentation of altered peptides that trigger an immune response [17, 18]. This process changes the shape of the antigen-binding cleft of the HLA molecule, which influences the repertoire of peptides presented to the immune system. Essentially, the drug modifies the normal set of peptides recognized by the immune system and, as a result, T-cells may react to the changed peptides, resulting in a drug hypersensitivity reaction.

High molecular weight therapeutic agents such as monoclonal antibodies (mAbs) often contain murine-derived structures which are recognized as foreign by the immune system, resulting in primarily type I (IgE and non-IgE mediated), cytokine release type, or type III (immune-complex-mediated) reactions [19]. Mixed reactions with both type I and cytokine release phenotypes may occur in the context of allergy to chemotherapeutic agents [20].

Unlike immune-mediated drug reactions, non-allergic reactions (previously called pseudoallergic or anaphylactoid reactions) are not associated with the production of antibodies or sensitized T cells but are often clinically indistinguishable from immune-mediated drug HSRs. During these reactions, the drug has the ability, via its chemistry or pharmacology, to directly stimulate the release or activation of inflammatory mediators, such as histamine, from mast cells and basophils. Non-steroidal anti-inflammatory drugs (NSAIDs), opioids, radiocontrast media, and angiotensin-converting enzyme (ACE) inhibitors are common causes of these non-allergic reactions [6, 21, 22].

Adverse reactions to vaccines are often reported, but clinically confirmed hypersensitivity to vaccines is rare, occurring at a rate of one per million vaccine doses for many vaccines [23–25]. Vaccine adverse reactions can occur to the microbial component of the vaccine or, more commonly, to an excipient in the vaccine preparation, such as egg protein, gelatin, or formaldehyde [26]. Assessment of a vaccine-associated reaction requires careful evaluation to determine whether the reaction is immunologically mediated, and whether testing or re-administration of the vaccine is indicated. The approach to the assessment and management of vaccine allergy differs from that of drug allergy and will be the topic of a future review.

## Risk factors

Certain patient- and drug-related factors are associated with an increased risk of developing drug allergy (Table 3) [27]. Drug allergy typically occurs in young and middle-aged adults and is more common in women. Historically, women are under-represented in drug trials, resulting in a lack of data to guide therapy in this population [28, 29]. Genetic polymorphisms and certain viral infections (Table 3) are also associated with an increased risk of immunologic reactions to drugs [27]. Topical and parenteral routes of administration, prolonged high or frequent doses, and large macromolecular drugs (e.g., insulin or horse antisera) or drugs that haptenate (bind to tissue or blood proteins and elicit an immune response), such as penicillin, are also associated with a greater likelihood of causing HSRs [27]. Atopic patients are not at increased risk for drug allergy, but they are at higher risk for serious allergic reactions [5, 27, 30–32]. A family history of drug allergy is not a known risk factor for a personal drug allergy.

**Table 3:** Risk factors for the development of drug allergy [27]

**Patient-related factors**: • Age: young/middle-aged adults > infants/elderly • Gender: Women > men • Genetic polymorphisms • HLA (a gene product of the MHC) • Drug metabolism • Viral infections: HIV, EBV, herpes viruses • Previous reaction to the drug
**Drug-related factors**: • High molecular weight compounds and hapten-forming drugs are more immunogenic • Route: topical > IV/intramuscular > oral • IV administration → more severe reactions • Dose: frequent/prolonged > single dose

EBV: Epstein-Barr virus; HIV: human immunodeficiency virus; HLA: human leukocyte antigen; IV: intravenous; MHC: major histocompatibility complex

## Diagnosis

The diagnosis of drug allergy requires a thorough history and the identification of physical findings and symptoms that are compatible with the characteristics and timing of drug-induced allergic reactions. Depending on the history and physical examination, diagnostic tests such as skin testing and drug challenges may be required [1, 5, 10, 27]. Therefore, if drug allergy is suspected, evaluation by an allergist experienced in these diagnostic procedures is recommended.

### History

Evaluation of the patient with a suspected drug allergy requires a detailed history, including: the timing and route of drug exposure, drug dosage, progression and characterization of signs and symptoms, treatment received and timeline for resolution of symptoms, prior prescription/non-prescription drugs taken by the patient, as well as previous and subsequent drug exposures and reactions [1, 5, 10, 27].

### Clinical presentation

In addition to the detailed history, a careful physical examination can help to define possible mechanisms underlying the reaction and guide subsequent investigations and diagnostic testing. Table 4 highlights some of the most common clinical manifestations of drug allergy and examples of causative drugs.

**Table 4:** Clinical manifestations of drug allergy [1, 22, 27]

Manifestation	Clinical Features	Examples of causative drugs
**Skin**		
Morbilliform drug eruption	• Diffuse, fine macules and papules • Evolve over days post drug initiation	Allopurinol, penicillins, cephalosporins, anticonvulsants, sulfonamides, mAbs
Urticaria, angioedema	• Onset within minutes to hours of drug administration • Potential for anaphylaxis • Often IgE-mediated	Antibiotics, ACE inhibitors, anticonvulsants, neuromuscular blocking agents, platinums, radiocontrast media, NSAIDs, opioids, mAbs
Fixed drug eruption	• Hyper-pigmented plaques that occur at the same site upon re-exposure to the culprit drug	Sulfonamide and tetracycline antibiotics, NSAIDs, ASA, sedatives, chemotherapeutic agents, anticonvulsants
**Hematologic**	• Hemolytic anemia, leukopenia, thrombocytopenia	Penicillin, sulfonamides, anticonvulsants, cephalosporins, quinine, heparin, thiazides, gold salts
**Hepatic**	• Hepatitis, cholestatic jaundice	Sulfonamides, phenothiazines, carbamazepine, erythromycin, anti-tuberculosis agents, allopurinol, gold
**Renal**	• Interstitial nephritis, glomerulonephritis	Penicillin, sulfonamides, allopurinol, PPIs, ACE inhibitors, NSAIDs
**Multi-organ**		
Anaphylaxis	• Urticaria/angioedema, bronchospasm, gastrointestinal symptoms, hypotension	Antibiotics, neuromuscular blocking agents, anesthetics, radiocontrast media, recombinant proteins (e.g., omalizumab)
Serum sickness	• Urticaria, arthralgias, fever	Heterologous antibodies, infliximab, allopurinol, thiazides, antibiotics (e.g., cefaclor), bupropion, mAbs
DILE	• Arthralgias, myalgias, fever, malaise	Hydralazine, procainamide, isoniazid, quinidine, minocycline, antibiotics, and anti–TNF-alpha agents
Vasculitis	• Cutaneous or visceral vasculitis	Sulfonamide antibiotics and diuretics, hydralazine, penicillamine, propylthiouracil, mAbs
SJS	• Fever, sore throat, fatigue, ocular involvement • Ulcers and other lesions on mucous membranes, particularly of the mouth and lips, as well as on truncal area	Sulfonamides, nevirapine, corticosteroids, anticonvulsants, NSAIDs (oxicams), allopurinol, phenytoin, carbamazepine, lamotrigine, barbiturates, psychotropic agents, pantoprazole, tramadol, mAbs
TEN	• Similar to SJS, but usually involves significant epidermal detachment Potentially life-threatening	Same as SJS
DRESS syndrome	• Cutaneous eruption, fever, eosinophilia, hepatic dysfunction, lymphadenopathy	Anticonvulsants, sulfonamides, minocycline, allopurinol, mAbs
AGEP	• Non-follicular sterile pustular rash over widespread erythema, fever and laboratory abnormalities	Antibiotics (penicillins, cephalosporins), antimycotics, other (diltiazem, antifungals, analgesics)

ACE: angiotensin-converting enzyme; AGEP: AGEP: acute generalized exanthematous pustulosis; ASA: acetylsalicylic acid; DILE: drug-induced lupus erythematosus; DRESS: drug rash with eosinophilia and systemic symptoms; IgE: immunoglobulin E; mAbs: monoclonal antibodies; NSAIDs: non-steroid anti-inflammatory drugs; PPIs: proton pump inhibitors; SJS: Stevens-Johnson syndrome; TEN: toxic epidermal necrolysis; TNF: tumour necrosis factor

The skin is the organ most frequently and prominently affected by drug-induced allergic reactions [1, 10, 22]. The most common cutaneous manifestation is a generalized morbilliform drug eruption (MDE), which is characterized by raised (papular), red (erythematous) lesions that appear within days to 3 weeks after drug exposure, with a generalized distribution. Lesions typically originate in the truncal area and eventually spread to the limbs. Urticaria (hives) and angioedema (swelling) are also common and can result from both IgE-mediated and non-IgE-mediated mechanisms. Compared with the adult population, the most likely cause of delayed maculopapular rashes and acute urticaria/angioedema in the pediatric population is a viral infection, and children with these presentations have a lower rate of true (IgE-mediated) drug allergy [33, 34].

Although skin reactions are the most common physical manifestation of drug-induced allergic reactions, many other organ systems may be involved, such as the renal, hepatic and hematologic systems (Table 4). Multi-organ reactions may also occur and include immediate reactions, such as anaphylaxis (see *Anaphylaxis* article in this supplement), but also delayed reactions, such as severe cutaneous adverse reaction (SCAR), serum sickness, drug-induced lupus erythematosus (DILE) and vasculitis (a heterogeneous group of disorders that are characterized by inflammatory destruction of blood vessels). SCAR are life-threatening and include: Stevens-Johnson syndrome (SJS) and toxic epidermal necrolysis (TEN), drug rash with eosinophilia and systemic symptoms (DRESS) syndrome and acute generalized exanthematous pustulosis (AGEP).

Serum sickness is an immune-complex-mediated reaction that presents with fever, lymphadenopathy, arthralgia, and cutaneous lesions. Serum sickness-like reactions are more common in children and tend to occur after infections or administration of some vaccines or drugs, such as penicillin [35]. However serum sickness-like reactions may also occur with newer mAbs that contain foreign murine components in the variable regions [36]. The exact mechanism of serum sickness-like reactions is poorly understood. The typical symptoms of DILE include sudden onset of fever and malaise; myalgia, arthralgia, and arthritis may also occur several weeks after drug initiation. In approximately 25% of cases, the skin may also be affected [1, 22]. Serum sickness and DILE are usually self-limited, with symptoms resolving spontaneously within a few weeks after discontinuation of the offending drug. Atypical symptoms, such as headache and chest, back or pelvic pain associated with acute fever and rigor, are suggestive of a cytokine release reaction to chemotherapeutic and biologic agents [19, 37].

Since the clinical manifestations of drug allergy are highly variable, it is important to exclude other conditions that may mimic drug-induced allergic reactions. Table 5 lists some of the conditions that should be considered in the differential diagnosis of drug allergy.

**Table 5:** Conditions to consider in the differential diagnosis of drug allergy [6]

IgE-mediated drug allergy (urticaria, angioedema, anaphylaxis) Non-IgE mediated reactions (MDE, DRESS syndrome, SJS, TEN)	
• Carcinoid syndrome	• Acute graft-versus-host disease
• Insect bites/stings	• Kawasaki disease
• Mastocytosis	• Still’s disease
• Asthma exacerbation	• Psoriasis
• Food allergy	• Insect bites/stings
• Scombroid fish poisoning	• Viral infection with exanthem
• Latex allergy	• Streptococcal infection
• Infection (EBV, hepatitis A, B, C, gastrointestinal parasites)	• Vasculitides
• Flare of chronic spontaneous urticaria/angioedema	• Cutaneous manifestation of connective tissue disease

DRESS: drug rash with eosinophilia and systemic symptoms; EBV: Epstein-Barr virus; IgE: immunoglobulin E; MDE: morbilliform drug eruption; SJS: Stevens-Johnson syndrome; TEN: toxic epidermal necrolysis

### Diagnostic tests

#### Immediate reactions

Skin testing procedures, such as skin prick tests (SPT) and intradermal tests (IDT; allergen is injected into the dermis) have been traditionally used to aid in the diagnosis of IgE-mediated (type I) reactions. However, the diagnostic accuracy of skin tests for most drugs is variable. Skin testing may be used in patients who are at high risk of anaphylaxis based on their reaction history, and potentially to overcome the nocebo effect (i.e., patients are more likely to experience an adverse effect if they expect or are worried about adverse effects) described in some individuals with a history of drug allergy [38]. Efforts are underway to standardize reproducible, non-irritating skin testing concentrations for drug allergy [39].

Serum-specific IgE tests are available for a limited number of drugs. However, these tests are costly, generally less sensitive and not more specific than skin tests. Furthermore, most of these in vitro tests are not adequately validated for drug allergy testing [1, 27]. Therefore, in most clinical settings, serum-specific IgE tests for medications are not used for the diagnosis of drug allergy.

#### Delayed reactions

Patch testing (PT) involves placing potential allergens (at non-irritant concentrations) on the patient’s skin for 48 h, and then assessing for reactions. Drug PT is useful for the diagnosis of various delayed (type IV) cutaneous reactions [1, 21, 22, 27, 40]. IDT with delayed reading can be performed with various non-irritating concentrations of sterile parenteral commercially manufactured preparations [41]. Similar to PT, these tests should not be performed at least 4 to 6 weeks after an acute reaction. The sensitivity of delayed IDT for antimicrobials ranges from 6.6–36.3% for MDE and 64–100% for DRESS syndrome [42, 43].

### Challenge

Drug challenge represents the gold-standard test to rule out an IgE-mediated ADR. More recently, direct drug challenge (with no prior skin tests) is used for the diagnosis of drug allergy, particularly in patients with a history of isolated, mild skin reactions after receiving beta-lactam antibiotics, such as penicillin [44–46]. The challenge can be performed in a single step (in a low-risk patient) or multiple steps. A graded challenge entails the administration of a test dose (typically 10% of one dose) of the medication, followed by a period of observation. If the patient tolerates the test dose, the remainder of the medication dose is administered, with another period of observation. Based on the clinical history of the reaction and patient-specific comorbidities that modulate the risk of anaphylaxis, challenges can be performed in a setting with rapid access to a resuscitation team. The challenge can be done as an “open label” procedure or in a blinded fashion with placebo control, depending on the patient and reaction history. Direct oral challenges without prior skin tests are increasingly being used to assess for amoxicillin and cephalosporin allergy in cases of benign, skin-limited reactions, including serum sickness-like reactions without bullous or vesicular lesions [44–51].

### Point-of-care tools

In adults, point-of-care, clinical history-based, risk prediction models have been generated to predict the risk of conducting direct oral challenges [52]. These models can help non-allergists and allergists quickly assess whether re-administration of penicillin is appropriate in a given patient. PEN-FAST is a novel, internally and externally validated penicillin allergy clinician decision rule that can identify low-risk penicillin allergies (Table 6) [46, 52, 53]. In patients with a reported penicillin allergy, a PEN-FAST score of < 3 is associated with a 96.7% negative predictive value [52].

**Table 6:** PEN-FAST penicillin allergy risk score

**PEN** — Penicillin allergy reported by patient *(if yes, proceed with assessment)*	
**F** — Five years or less since reaction —2 points	
**A** — Anaphylaxis or angioedema OR	
**S** — Severe cutaneous adverse reaction —2 points	
**T** — Treatment required for reaction —1 point	
**Interpretation**: 0: Very low risk of positive penicillin allergy test < 1% (< 1 in 100 patients reporting penicillin allergy) 1–2: Low risk of positive penicillin allergy test ∼ 5% (1 in 20 patients) 3: Moderate risk of positive penicillin allergy test ∼ 20% (1 in 5 patients) 4–5: High risk of positive penicillin allergy test ∼ 50% (1 in 2 patients)	

Although PEN-FAST has not been shown to be useful in children [54], clinical pediatric prediction tools, including an electronic algorithm developed in Canada, are in the process of validation [55, 56]. The algorithm has been adapted into a clinical decision support tool that may help non-allergists risk stratify penicillin allergy (see “Firstline—Clinical Decisions”) [57]. Other point-of-care guides include: the Institut national d’excellence en santé et en services sociaux (INESSS) decision support tool [58] and a review by Shenoy et al. that provides guidance on risk stratification [59]. PEN-FAST was recently successfully adapted for sulfonamide antibiotic allergy (SULF-FAST) [60]. SULF-FAST can identify individuals at low-risk for a true (IgE-mediated) allergy who could proceed to an oral challenge as a delabelling strategy.

### Laboratory tests

The measurement of tryptase levels (within 3 h of a reaction) has proved useful in confirming acute IgE-mediated reactions, particularly anaphylaxis; however, negative results do not rule out acute allergic reactions. A complete blood count can help diagnose hemolytic (type II) drug-induced reactions, such as hemolytic anemia, thrombocytopenia, or neutropenia. Hemolytic anemia may also be confirmed with a positive direct and/or indirect Coombs’ test (used to examine for the presence of antibodies on red blood cell membranes) [1, 22, 27].

Studies have examined the potential role of the basophil activation test (the quantification of basophil activation by flow cytometry) in the diagnosis of drug allergy, since basophils are involved in both immune-mediated and non-immune-mediated reactions. Although some evidence suggests that the test is useful for evaluating possible allergies to beta-lactam antibiotics, NSAIDs and muscle relaxants, further confirmatory studies are needed before it is widely accepted as a diagnostic tool [1, 61, 62].

Lymphocyte transformation assays can play a role in assessing delayed, T-cell–mediated drug reactions [63, 64]. Some specialized centers are developing new laboratory tools which examine cytokine production from isolated patient T cells (i.e., interferon-gamma [IFN-γ] release enzyme-linked immunospot [ELISpot]) to help evaluate drug causality. At this time, however, their use is reserved for research purposes only [43, 65, 66]. Also, there are currently no validated commercial assays for these tests in North America.

## Management of common drug allergies

The most effective strategy for the management of drug allergy is avoidance or discontinuation of the offending drug. When available, alternative medications with unrelated chemical structures should be substituted. Cross-reactivity among drugs should also be taken into consideration when choosing alternative agents [1, 22]. In cases where there is a definite medical need for a particular drug (with no acceptable alternative) and the clinical history is indicative of an IgE-mediated reaction, a procedure to induce temporary drug tolerance (also referred to as drug desensitization) may be considered.

Additional therapy for drug HSRs is largely supportive and symptomatic. For example, topical corticosteroids and oral antihistamines may improve cutaneous symptoms. In the event of anaphylaxis, the treatment of choice is epinephrine, which is administered by intramuscular injection into the lateral thigh (see the *Anaphylaxis* article in this supplement). Systemic corticosteroids and/or immunomodulators may also be used to treat severe systemic reactions [67], but should never be given prior to, or replace, epinephrine in the treatment of anaphylaxis. Severe drug reactions, such as SJS and TEN, are best treated in an intensive care or burn unit setting [1, 22, 68]. Strategies for the management of some of the most common drug allergies are discussed below.

### Penicillin

Penicillin and its derivatives are the most frequent drug allergies, affecting approximately 10% of patients [69]. For patients with confirmed penicillin allergy, treatment is best limited to non-penicillin agents. Reassessment for continued allergy should occur periodically as penicillin sensitization wanes over time [70]. Carbapenems (e.g., imipenem) do not exhibit a significant degree of cross-reactivity with penicillin and may be administered [71–74]. Monobactams, such as aztreonam, are generally well tolerated by patients with penicillin allergy, except if they had an allergic reaction to ceftazidime [75–77]. Different R-chain cephalosporins may also be considered since the degree of cross-reactivity with these agents and penicillin has been shown to be lower than with same R-chain agents (see following *Cephalosporin* section) [1, 74, 78].

Diagnosis of the patient with penicillin allergy should include penicillin allergy assessment and confirmation. Studies have shown that among patients who report a penicillin allergy, more than 80% have negative skin testing [79]. Approximately 96–99% of patients labelled with a low-risk penicillin allergy have negative penicillin oral challenge responses (i.e., have no reaction when challenged) and can safely receive cephalosporins and other beta-lactam agents [1, 80–83]. Furthermore, 90% of patients tolerate penicillin upon further evaluation [8].

Patients with a suspected allergy to penicillin may be prescribed alternate antimicrobials that may be less effective, more toxic or more expensive. In fact, a penicillin allergy label has been associated with negative clinical and administrative outcomes, including more hospitalizations, increased antibiotic-resistant infections, greater medical costs, and increased mortality [84–91]. As a result, there has been increased focus to remove the label of ‘drug allergy’, particularly to penicillin [92–95]. Multidisciplinary programs, with involvement of antimicrobial stewardship groups, allergists and pharmacists, have been shown to improve patient-related outcomes and reduce healthcare costs [96]. Point-of-care clinical decision rules, like the PEN-FAST score [52], may augment penicillin allergy delabelling strategies.

If penicillin is deemed absolutely necessary in a high-risk penicillin-allergic patient that presented with an IgE-mediated reaction, desensitization (discussed later) should be considered, and the procedure should only be performed in-hospital under medical supervision.

### Cephalosporins

The most common reactions to cephalosporins are morbilliform rashes; urticaria is less common and anaphylaxis is rare [78]. Demonstrated sensitization to penicillin is associated with a higher likelihood of allergic reactions to first-generation cephalosporins (about 2%); however, this cross-reactivity was based on skin testing and was not clinically confirmed by challenge [97]. In fact, Macy and Ngor found the incidence of clinical reactions to first-generation cephalosporins to be the same as to sulphonamide antibiotics in penicillin-intolerant patients [98]. In penicillin-allergic patients, it may be advisable to avoid first-generation cephalosporins unless skin testing and challenge to an appropriate cephalosporin is negative. In cephalosporin-allergic subjects, there is limited cross-reactivity on immunological testing between second- and third-generation cephalosporins and penicillins, especially amino-penicillins, but this has not necessarily indicated clinical reactivity [99]. There is a role for testing with the proposed antibiotic to be used in therapy, by graded challenge, possibly preceded by skin testing. More recently, it was reported that in children with non-severe, skin-limited symptoms during cephalosporin treatment, a direct oral challenge is a safe and appropriate diagnostic strategy [45]. If testing is positive and no alternative drug exists in a patient with severe IgE-mediated reactions, induction of drug tolerance procedures may be attempted [1, 6].

### Macrolides

By virtue of their widespread use, allergies to macrolide antibiotics are commonly reported [100]. At present, no validated or standardized skin or serum test is available for macrolide allergy testing. Therefore, oral challenge is recommended if the history of the index reaction is low risk (i.e., a mild reaction entailing symptoms that do not meet the criteria for a drug allergy). If the reaction was severe, then avoidance is generally recommended.

### Sulfonamides

Sulfonamide antibiotics are another common cause of drug-induced allergic reactions, and can be associated with severe delayed cutaneous eruptions, such as SJS and TEN. Trimethoprim-sulfamethoxazole (TMP-SMX) is the drug of choice for the prophylaxis and treatment of a number of opportunistic infections and, therefore, many human immunodeficiency virus (HIV)-positive patients with a history of reacting to sulfonamides still require treatment with this antibiotic. Induction of drug tolerance procedures can be used to safely administer TMP-SMX to patients with a history of severe IgE-mediated allergy to the antibiotic. For patients with a history of benign cutaneous reactions, including morbilliform rashes or urticaria that occurred more than 5 years ago, a single-step drug challenge with TMP-SMX can be performed when there is a need to delabel a sulfonamide antibiotic allergy [8].

Since the chemical structure of non-antibiotic sulfonamides (e.g., thiazide diuretics, some NSAIDs and anticonvulsants) varies from sulfonamide antibiotics, these agents are not expected to cross-react, and can generally be safely administered to patients with a history of allergy to sulfonamide antibiotics. An exception is sulfasalazine, which is metabolized to sulfapyridine. This metabolite resembles the antigenic structure of sulfamethoxazole [1, 101–103] and should be avoided in the setting of a confirmed sulfonamide antibiotic allergy.

### Fluoroquinolones

Although IgE-mediated allergy to fluoroquinolones is possible, these drugs can directly activate mast cells and cause symptoms that mimic anaphylaxis [104]. At present, no validated skin test is available for this class of medications. As such, depending on the index reaction, a graded challenge with either the suspect fluoroquinolone (in the setting of a remote, mild reaction) or with an alternate medication from the same class is recommended [8].

### Radiocontrast media

Radiocontrast media (RCM) is associated with both IgE-allergic and non-IgE-mediated reactions. The incidence of reactions to RCM, including severe, life-threatening reactions, is lower with non-ionic versus ionic agents [105]. Non IgE-mediated reactions to RCM have classically been managed with pretreatment regimens that include oral corticosteroids and H1-antihistamines. However, the evidence in favour of this practice is equivocal [106]. Low osmolarity agents should also be used in such circumstances [1, 6]. Pretreatment with antihistamines is permissible in highly anxious patients, but steroids should be avoided [106].

### Local anesthetics

IgE-mediated allergic reactions to local anesthetics (e.g., procaine [Novocaine], lidocaine) are extremely rare; reactions are usually due to other ingredients in the medication, such as preservatives (e.g., metabisulfites or parabens). Patients may also experience Type A adverse effects to adrenaline, which is sometimes combined with local anesthetic injections [107]. However, if the reaction history is consistent with a possible immediate, IgE-mediated (type I) reaction, skin testing followed by graded challenge tests using epinephrine-free, preservative-free local anesthetics may be utilized [1].

### General anesthetics

Although rare, anaphylaxis may occur in patients receiving medications for general anesthesia. The investigation of severe reactions during general anesthesia is particularly challenging given that the patient is often exposed to many co-administered drugs and agents. Reactions during general anesthesia can be due to neuromuscular blocking agents [108], but have also been associated with IV anesthetics (e.g., propofol, thiopentone, etomidate). It is important to consider other agents in the perioperative context when assessing for general anesthetic allergy, including antibiotics, NSAIDs, chlorhexidine (present in alcohol swabs and wipes), opioids, and latex. The incidence of chlorhexidine allergy has increased over time, as has the incidence of antibiotic allergies, especially cefazolin, which is widely used in perioperative anaphylaxis [109–111]. Opioids may be confounders as they can either mimic or amplify these reactions by directly activating mast cells via the MRGPRX2 receptor. There are no reported cases of allergy to inhaled anesthetics. Assessment by an allergist is important for confirming the clinical diagnosis of allergy to general anesthetic medications, identifying likely causative agents, as well as alternative agents that may be used safely in the future [112].

### Acetylsalicylic acid/NSAIDs

Acetylsalicylic acid (ASA) and NSAIDs can cause both IgE-mediated and non-IgE mediated reactions, including exacerbations of underlying respiratory diseases, urticaria, angioedema, and anaphylaxis. Patients with underlying chronic respiratory diseases, such as asthma, rhinitis, and sinusitis, may react to ASA and NSAIDs that inhibit cyclooxygenase-1 (COX-1). The major clinical phenotypes of NSAID-induced reactions can be categorized into acute and delayed reactions, with further categorization based on symptoms (Table 7) [113]. In some patients, clinical history alone might be sufficient to establish the diagnosis of a specific type of NSAID hypersensitivity, whereas in other cases, oral provocation challenges are necessary to confirm the diagnosis. Moreover, classification of the type of cutaneous reaction is critical for proper management. For example, in patients with single NSAID-induced reactions (where the patient reacts to one specific NSAID but can tolerate others), chemically non-related COX-1 inhibitors can be safely used [113].

**Table 7:** Classification of NSAID-induced reactions [113]

Type of reaction	Symptoms	Comorbidity	Single vs. multiple NSAID(s)
Acute	Urticaria/angioedema	Chronic urticaria	Multiple
	Urticaria/angioedema	None known	Single or multiple
	Anaphylaxis	Atopy	Single
	Asthma/rhinitis/sinusitis flare (NSAID-exacerbated respiratory disease)	Asthma/nasal polyps	Multiple
Delayed (more than 24 h after exposure)	Morbilliform drug eruptions	None known	Single or multiple
	Severe cutaneous reaction	None known	Single or multiple
	Organ dysfunction (pneumonitis, aseptic meningitis, nephritis)	None known	Single or multiple

NSAID: non-steroidal anti-inflammatory drugs

There are no standardized skin tests for the diagnosis of NSAID allergy. Diagnosis should be established by challenge, preferably in a hospital setting. One study found that up to 20% of pediatric patients will react during challenge, and 20% of those with a negative challenge may still react upon subsequent treatment (primarily older children) [114].

The management of patients with NSAID-exacerbated respiratory disease involves avoidance of aspirin and NSAIDs and aggressive treatment of the underlying respiratory disorder. Selective COX-2 inhibitors rarely cause reactions, and can typically be taken safely by patients with ASA/NSAID allergy. An induction of drug tolerance procedure to aspirin (also known as aspirin desensitization) may also be considered in aspirin-exacerbated respiratory diseases [1].

Patients with chronic urticaria/angioedema generally tolerate COX-2 inhibitors, but may experience exacerbations of urticaria/angioedema with NSAIDs that inhibit COX-1. IgE-mediated allergic reactions to NSAIDs are usually drug specific and, therefore, patients experiencing these reactions are often able to tolerate other NSAIDS [1].

### Monoclonal antibodies (mAbs)

mAbs are proteins with inherent immunogenicity. HSRs to mAbs, which can range in severity from mild to life-threatening, represent an escalating clinical problem since these biologics are increasingly being used for the treatment of various inflammatory, autoimmune, and malignant diseases [115, 116]. The risk of developing reactions to mAbs depends on the humanization of the mAb (i.e., fully human mAbs are considered less immunogenic than humanized or chimeric mAbs, which contain variable amounts of sequences of mouse origin), the type of Ig elicited (i.e., IgE vs. IgG), the activation of complement, and the presence of adjuvants and excipients [115]. Most mAb-related adverse reactions are due to the infusion (“infusion reactions”) or cytokine release and lack immune specificity (e.g., fever, rigors, chills, headache, chest/back pain, increased blood pressure, gastrointestinal symptoms) [19]. However, immune-specific HSRs may also occur, and these can overlap with non-immune mechanisms leading to complex clinical presentations [19, 117–119]. It should be noted that infusion reactions due to cytokine release typically occur upon first administration of the mAb and generally wane rapidly with subsequent exposures. Although there is overlap in symptoms between infusion reactions and IgE-mediated reactions, infusion reactions are more common, occur predictably, often with initial doses, and improve with antihistamine premedication and infusion rate reduction [119].

HSRs to mAbs are classified as immediate (onset within a few hours of infusion) and non-immediate (onset from a few hours to 14 days after infusion). The reactions can be systemic or local (at the injection site). Immediate HSRs, such as urticaria, bronchospasm, and multi-organ anaphylaxis, are mediated by IgE (mast cell/basophil activation) or IgG (basophil activation) [19]. IgE-mediated reactions to mAbs typically occur after previously well-tolerated exposures because sensitization has to take place before a reaction can develop. However, IgE-mediated reactions have been noted during the very first administration of cetuximab (a chimeric mAb used in the treatment of colorectal, lung, skin, and head and neck cancers) due to pre-existing IgE antibodies directed against an oligosaccharide (i.e., galactaose-alpha-1,3-galactose [alpha-gal]) present on this mAb [120, 121]. In IgE-mediated reactions, skin tests may be positive and/or tryptase may be elevated at the time of the reaction.

The most common manifestation of a non-immediate HSR to mAbs is a serum sickness-like reaction with vasculitic manifestations (e.g., fever, malaise, arthralgia/arthritis, jaw pain or tightness, erythematous or urticarial skin eruption, purpura, and conjunctival hyperemia) that typically appears 5 to 7 days after the infusion [122]. Maculopapular exanthema is another delayed reaction that has been noted with infliximab and abciximab. Rare, non-immediate reactions, such as symmetrical drug-related intertriginous and flexural exanthema (SDRIFE), SJS and TEN, have also been attributed to mAbs [122].

The management of mAb HSRs is still evolving, and evidence regarding the value of skin testing and IDT is expanding. For some mAbs, these tests have shown positive results, suggesting that reactions were IgE-mediated [19]. When severe HSRs to mAbs occur, an alternate drug should be given whenever possible. For example, panitumumab can replace cetuximab in patients with allergic reactions mediated by IgE antibodies to alpha-gal [123]. Like other drugs, desensitization is only indicated when the mAb is considered first-line therapy and there are no acceptable alternatives. When designed appropriately, desensitization protocols have proven successful in addressing both immune- and non-immune-mediated reactions. In these protocols, the rate of the mAb infusion is adjusted according to the severity of the initial hypersensitivity event, eventual breakthrough reactions during each desensitization course, and body weight (in pediatric patients) [124]. Desensitization is contraindicated in severe delayed HSRs, including serum sickness-like reactions.

Premedication may be an adjunct to desensitization, and should be tailored to the clinical characteristics of the index reaction. Depending on the index reaction and patient characteristics, premedication may include H1 or H2 antihistamines, montelukast, acetaminophen, antiemetics, and/or corticosteroids [19].

### Chemotherapeutic agents

HSRs to chemotherapy may prevent patients from receiving the most effective therapy. The incidence of HSRs to antineoplastic agents can increase with the number of treatments administered [125]. All parenteral chemotherapeutic agents have the potential to cause infusion-related reactions which may occur during the first or second infusion. These reactions vary in severity and involve one or multiple organs. The typical manifestations are flushing and chest pressure or tightness.

Infusion reactions to chemotherapy usually respond to premedication and/or slowing of the infusion rate. Premedication helps to prevent and/or reduce the severity of the HSR, but does not prevent anaphylaxis in most cases. Without premedication, HSRs can occur in up to 42% of patients, depending on the type of chemotherapeutic agent [20]. If the reaction is limited to mild or moderate symptoms, the drug infusion should be temporarily stopped and assessment of the airways, breathing, and circulation should be performed. Rechallenge is often possible after symptomatic treatment; restarting the drug at a slower infusion rate may allow treatment continuation with close monitoring. Similar to other drugs, HSRs to chemotherapies include immediate ( IgE- and non-IgE-mediated) and delayed reactions, and management involves switching the culprit agent to an alternative suitable chemotherapy or considering desensitization to maintain the first-line therapy [20].

Some clinical manifestations post chemotherapy exposure are not considered HSRs. For example, many patients experience a variety of toxic skin reactions, such as desquamative rash, hand-foot syndrome, and plaque-like erythrodysesthesia [126, 127]. Stopping the causal agent will lead to the resolution of cutaneous lesions.

## Desensitization

Unlike a drug challenge, which is used to rule out an allergy, a drug tolerance-induction procedure is undertaken when there is a confirmed allergy. Induction of drug tolerance procedures temporarily modify a patient’s immunologic or non-immunologic response to a drug through the administration of incremental doses of the drug. Most regimens begin with a very dilute concentration of the drug, and the dose is doubled every 15 to 20 min until a full therapeutic dose has been administered after 3 to 8 h. Drug tolerance is usually maintained only as long as the drug is administered; the procedure needs to be repeated in the future if the patient requires the drug again after finishing a prior therapeutic course. Drug tolerance-induction procedures should only be performed by experienced personnel in facilities with resuscitative equipment readily available [1, 128, 129].

## Prevention of future reactions

Prevention of future reactions is an essential part of patient management. The patient should be provided with written information about which drugs to avoid (including over-the-counter medications). The drugs should be highlighted in the hospital notes and within electronic records (where available), and the patient’s family physician and pharmacist should be informed of the drug allergy. Engraved allergy bracelets/necklaces, such as those provided by Medic Alert, should also be considered, particularly if the patient has a history of severe drug-induced allergic reactions [27].

## Conclusions

Drug allergy is a common clinical problem; assessment by an allergist is important for appropriate diagnosis and management of the condition. Diagnosis relies on a careful history and physical examination and, in some instances, skin or laboratory testing and graded challenges may be required. In select groups of patients, especially children with beta-lactam allergy, direct oral challenges may be appropriate. The mainstay of treatment for drug allergy is avoidance of the offending drug. When available, alternative medications with unrelated chemical structures should be substituted. Cross-reactivity among drugs should be taken into consideration when choosing alternative medications. If a particular drug to which the patient is allergic is indicated and there is no suitable alternative, induction of drug tolerance procedures may be considered.

## Data Availability

Not applicable.

## Abbreviations

ACE: Angiotensin-converting enzyme
ADR: Adverse drug reaction
AGEP: Acute generalized exanthematous pustulosis
alpha-gal: Galactaose-alpha-1,3-galactose
ASA: Acetylsalicylic acid
APCs: Antigen-presenting cells
COX-1: Cyclooxygenase-1
COX-2: Cyclooxygenase-2
DILE: Drug-induced lupus erythematosus
DRESS: Drug rash with eosinophilia and systemic symptoms
EBV: Epstein-Barr virus
ELISpot: Enzyme-linked immunospot
HLA: Human leukocyte antigen
HIV: Human immunodeficiency virus
HSR: Hypersensitivity reaction
IDT: Intradermal testing
IgE: Immunoglobulin E
IgG: Immunoglobulin G
IgM: Immunoglobulin M
INESSS: Institut national d’excellence en santé et en services sociaux
IFN-γ: Interferon-gamma
IV: Intravenous
mAbs: Monoclonal antibodies
MDE: Morbilliform drug eruption
MHC: Major histocompatibility complex
MRGPRX2: Mas-related G protein-coupled receptor X2
NSAIDs: Non-steroidal anti-inflammatory drugs
PT: Patch testing
RCM: Radiocontrast media
SCAR: Severe cutaneous adverse reaction
SDRIFE: Symmetrical drug-related intertriginous and flexural exanthema
SPT: Skin prick testing
SJS: Stevens-Johnson syndrome
TEN: Toxic epidermal necrolysis
TMP-SMX: Trimethoprim-sulfamethoxazole
TNF: Tumour necrosis factor
